# Soluble RAGE attenuates AngII-induced endothelial hyperpermeability by disrupting HMGB1-mediated crosstalk between AT1R and RAGE

**DOI:** 10.1038/s12276-019-0312-5

**Published:** 2019-09-27

**Authors:** Jisu Jeong, Jiye Lee, Juyeon Lim, Soyoung Cho, Soyoung An, Myungeun Lee, Nara Yoon, Miran Seo, Soyeon Lim, Sungha Park

**Affiliations:** 1 Graduate Program in Science for Aging, Yonsei University, Seoul, 120-752 Korea; 2 Integrative Research Center for Cerebrovascular and Cardiovascular Diseases, Yonsei University College of Medicine, Seoul, 120-752 Korea; 3 Department of Pathology, The Catholic University of Korea, Incheon St. Mary’s Hospital, Incheon, Korea; 4 Institute for Bio-Medical Convergence, College of Medicine, Catholic Kwandong University, Gangneung, Gangwon-do 25601 Korea; 5 Cardiovascular Research Institute, Division of Cardiology, Yonsei University College of Medicine, Seoul, 120-752 Korea

**Keywords:** Stress signalling, Senescence

## Abstract

Increased endothelial permeability, one of the earliest signs of endothelial dysfunction, is associated with the development of cardiovascular diseases such as hypertension and atherosclerosis. Recent studies suggest that the receptor for advanced glycation end products (RAGE) regulates endothelial permeability in inflammation. In the present study, we investigated the regulatory mechanism of RAGE in endothelial hyperpermeability induced by angiotensin II (Ang II), a well-known inflammatory mediator, and the potential therapeutic effect of soluble RAGE (sRAGE), a decoy receptor for RAGE ligands. For in vitro studies, Ang II-treated human umbilical vein endothelial cells (HUVECs) were treated with siRNA specific to either RAGE or sRAGE to disrupt RAGE-mediated signaling. Endothelial permeability was estimated using FITC-labeled dextran 40 and a resistance meter. To evaluate intercellular junction disruption, VE-cadherin expression was examined by western blotting and immunocytochemistry. Ang II increased the expression of the Ang II type 1 receptor (AT1R) and RAGE, and this increase was inhibited by sRAGE. sRAGE prevented Ang II-induced VE-cadherin disruption in HUVECs. For in vivo studies, Ang II-infused, atherosclerosis-prone apolipoprotein E knockout mice were utilized. Endothelial permeability was assessed by Evans blue staining of the aorta. Ang II increased endothelial barrier permeability, and this effect was significantly attenuated by sRAGE. Our data demonstrate that blockade of RAGE signaling using sRAGE attenuates Ang II-induced endothelial barrier permeability in vitro and in vivo and indicate the therapeutic potential of sRAGE in controlling vascular permeability under pathological conditions.

## Cardiovascular disease: Decoy protein improves blood vessel function

A decoy version of a protein involved in regulating the leakiness of blood vessels can help ameliorate vascular problems that lead to high blood pressure and plaque deposition in the arteries. A team from South Korea led by Soyeon Lim from Catholic Kwandong University in Gangneung and Sungha Park from Yonsei University College of Medicine in Seoul induced hyper-permeability in both human vein cells and atherosclerosis-prone mice. They then blocked signaling through a membrane-bound protein called RAGE, a receptor that helps boost vessel permeability by using a soluble version of this same protein. In both the human cells and mouse models, this free-floating RAGE bound and blocked the receptor’s normal activator, leading to suppressed permeability and improved function of the blood vessel lining. This decoy strategy holds therapeutic promise for people prone to cardiovascular disease.

## Introduction

Endothelial cells play a critical role in maintaining the homeostasis of blood vessels. Regulation of the endothelial barrier is important for maintaining the integrity of cell-to-cell adhesion because increased endothelial permeability can lead to endothelial dysfunction, a common prelude to the development of vascular diseases such as atherosclerosis^1^. Endothelial hyperpermeability is also a significant problem in a number of conditions, such as vascular inflammation, ischemia–reperfusion injury, diabetes, and thrombosis and cancer^2^. Excessive inflammatory stimuli, such as transforming growth factor-beta (TGF-β), thrombin, and tumor necrosis factor-alpha (TNF-α), can trigger actin cytoskeleton reorganization via such events as filamentous actin (F-actin) disruption, stress fiber formation, and cytoskeletal contraction, leading to transendothelial flux via the widened intercellular space^2,3^. Since reduced availability of nitric oxide due to high oxidative stress and inflammation can also cause endothelial hyperpermeability^4,5^, angiotensin II (Ang II) can be another key modulator of endothelial hyperpermeability, because the renin–angiotensin system (RAS) can lead to both high oxidative stress and inflammation, causing abnormal NO metabolism^6^.

Ang II is an important vasoconstrictor in the RAS and exerts multiple functional effects on endothelial cells, including excessive oxidative stress, vascular inflammation, thrombosis, and remodeling of the actin cytoskeleton^7^. Although Ang II is known to induce endothelial hyperpermeability^8^, and previous studies have shown that Rac and Rho kinase mediate Ang II-induced cytoskeletal remodeling^9,10^, the underlying mechanisms by which Ang II induces endothelial hyperpermeability have not been completely elucidated. The biological action of Ang II is mainly mediated by the Ang II type 1 receptor (AT1R), and recent studies have suggested a link between AT1R and the receptor for advanced glycation end products (RAGE) as an important signaling pathway in vascular smooth muscle cells and podocytes^11,12^.

RAGE is a multiligand signal transduction receptor that recognizes various endogenous ligands, such as high mobility group box 1 (HMGB1), advanced glycation end products (AGE), macrophage-1 antigen (Mac-1), and S100/calgranulins, and plays an important role in inducing inflammatory reactions through the activation of protein kinase C (PKC)/phosphoinositide 3-kinase (PI3K)/Akt, Janus kinase/signal transducer and activator of transcription (JAK/STAT), mitogen-activated protein kinase (MAPK), extracellular signal-regulated kinase (ERK), and Src/RhoA/Cdc42, leading to the activation of transcription factors such as NF-κB and Egr1^13^. Regarding the role of RAGE signaling in endothelial dysfunction, studies showed that AGE increased endothelial permeability through the RAGE/Rho signaling pathway or Src phosphorylation in human umbilical vein endothelial cells (HUVECs)^14,15^ and that AGE-induced Src phosphorylation and barrier dysfunction were inhibited in the pulmonary microvascular endothelial cells of RAGE knockout mice^14^.

Although these findings provide circumstantial evidence of possible crosstalk between AT1R-mediated signaling and RAGE-mediated signaling in Ang II-induced endothelial hyperpermeability, this link has not been empirically examined, and the underlying mechanism remains elusive. Therefore, in the present study, we investigated the regulatory mechanism of RAGE in Ang II-induced endothelial hyperpermeability and the potential therapeutic effect of soluble RAGE (sRAGE), a decoy receptor for RAGE ligands.

## Materials and methods

### Chemicals and antibodies

Purified human soluble RAGE-Fc fusion protein (sRAGE) was purchased from Y-biologics (Daejeon, Republic of Korea). Ang II, losartan, caffeic acid phenethyl ester (CAPE), and fluorescein isothiocyanate (FITC)-labeled dextran 40 were obtained from Sigma-Aldrich (St. Louis, MO, USA). Purified anti-HMGB1 antibody (651402) was purchased from BioLegend (San Diego, CA, USA). Anti-AT1R antibody (AAR-011) was purchased from Alomone Labs (Hadassah Ein Kerem, Israel). Anti-RAGE (ab3611), anti-mDia1 (ab129167), and anti-HMGB1 (ab18256) antibodies were purchased from Abcam (Cambridge, UK). Anti-Src (2109s), anti-phospho-Src (6943s, Tyr416), anti-β-catenin (8480s), anti-phospho β-catenin (5651s, Ser522), and anti-phospho-NF-κB (3033s, Ser536) antibodies were obtained from Cell Signaling Technology (Danvers, MA, USA). Anti-phospho-VE-cadherin (LS-C357312, Tyr731) was purchased from LifeSpan Biosciences (Seattle, WA, USA). Anti-VE-cadherin (sc9989), anti-NF-κB (sc8008), anti-β-actin (sc47778), and anti-GAPDH (sc32233) antibodies were purchased from Santa Cruz Biotechnology (Dallas, TX, USA).

### Cell culture

HUVECs were purchased from Lonza and maintained in endothelial growth medium-2 (EGM-2, Lonza, Basel, Switzerland) supplemented with 2% (v/v) fetal bovine serum (FBS) at 37 °C in a humidified atmosphere containing 5% CO_2_. HUVECs were cultured in 1.5% gelatin-coated cell culture dishes, and cells at passages 4–8 were used in the experiments.

### Permeability assay

HUVECs were grown in the upper chambers of 24-well Transwell inserts (0.4 μm pore size; Corning, NY, USA) until a confluent monolayer was formed. For endothelial permeability assays, HUVECs were starved in serum-free EBM-2 for 3 h. After starvation, the upper and lower chambers were pretreated with sRAGE for 1 h and then with Ang II for 4 h. Subsequently, fluorescein isothiocyanate (FITC)-labeled dextran 40 (final concentration of 10 mg/ml in basal medium) was added as a tracer to the upper chamber, and cells were incubated for 1 h at 37 °C. After incubation, the medium in the lower chamber was collected into a black 96-well plate, and fluorescence was measured with a fluorescence plate reader (Varioskan Flash; Thermo Fisher Scientific, Waltham, MA, USA) at 485 nm excitation and 530 nm emission wavelengths. The permeability of the endothelial monolayer was evaluated by calculating the permeability coefficient of dextran by the following formula: Pd = [*A*]/*t* × 1/*A* × *V*/[*L*], where [*A*] is the dextran concentration in the bottom chamber, *t* is the time in seconds, *A* is the area of the membrane (in cm^2^), *V* is the volume of the bottom chamber, and [*L*] is the dextran concentration in the upper chamber^14^.

### Transendothelial electrical resistance

The transendothelial electrical resistance (TEER) of the HUVEC monolayer was determined using an STX2 electrode and EVOM^2^ meter (World Precision Instruments, Sarasota, FL, USA) according to the manufacturer’s instructions. HUVECs were seeded in the upper chambers of a 12-well Transwell insert (0.4 μm pore size) and grown to confluence. The resistance values of multiple Transwell inserts in each experimental group were measured sequentially, and the mean was expressed in common units (ohm cm^2^) after the value of a blank, cell-free insert was subtracted. The values were normalized to that of untreated control cells before stimulation.

### Immunocytochemistry

HUVECs were plated in four-well glass chamber slides and cultured to confluence. After sequential treatment with the indicated stimulant, cells were washed in phosphate-buffered saline (PBS) three times, and were then fixed with 4% paraformaldehyde for 10 min. Cells were washed again with PBS, followed by blocking with 0.5% bovine serum albumin (BSA) at room temperature and incubation with the appropriate mouse monoclonal VE-cadherin antibody overnight at 4 °C. After three washes with PBS, cells were incubated with FITC-conjugated goat anti-mouse antibody at room temperature for 1 h in the dark. Cells were mounted using DAPI-containing mounting medium (Santa Cruz Biotechnology). Immunoreactivity signals were visualized by confocal laser scanning microscopy (LSM780; Carl Zeiss, Dresden, Germany).

### RNA interference

A TriFECTa DsiRNA Kit for Diaphanous-related formin-1 (mDia1) was purchased from IDT (Coralville, IA, USA). Scrambled and RAGE siRNAs (siRNA no. 1003394) were purchased from Bioneer (Daejeon, Republic of Korea). Briefly, cells at 60–80% confluence were transfected with 200 nM RAGE siRNA, scrambled siRNA (negative control), or 20 nM *mDia1* siRNA using RNAiMax (Invitrogen, Carlsbad, CA, USA) according to the manufacturer’s instructions. After 4 h of transfection, the medium was exchanged with fresh medium.

### Western blot analysis

HUVECs were lysed in RIPA buffer (Biosesang, Gyenoggi-do, Republic of Korea) containing a mixture of protease inhibitors (Thermo Fisher Scientific, Waltham, MA, USA) and centrifuged to extract total protein. Protein concentrations were determined using a BCA protein assay kit (Sigma-Aldrich, St. Louis, MO, USA) according to the manufacturer’s instructions, and 25 µg of protein was analyzed by 8% sodium dodecyl sulfate-polyacrylamide gel electrophoresis (SDS-PAGE) (Bio-Rad, Hercules, CA, USA). Membranes with transferred proteins were blocked with 5% skim milk (BD Biosciences, San Jose, CA, USA) for 1 h and incubated overnight at 4 °C with antibodies against the following proteins: AT1R, RAGE, mDia1, Src, phospho-Src, β-catenin, phospho-β-catenin, VE-cadherin, phospho-VE-cadherin, HMGB1, NF-κB, phospho-NF-κB, β-actin, and GAPDH. The relative intensities of protein bands were analyzed using ImageJ software (National Institutes of Health, Bethesda, MD, USA).

### Reverse transcription polymerase chain reaction

RNA was isolated from cells using a Ribospin kit (GeneAll, Seoul, Republic of Korea) according to the manufacturer’s instructions. Single-stranded complementary DNA (cDNA) was synthesized from 1 μg of total RNA with oligo-deoxythymidine (dT) primers using reverse transcriptase (Bio-Rad). The RAGE primer set was purchased from Bioneer (p202745). Additional primer sets were purchased from IDT and validated. The following primer sequences were used: 5′-CAC CAT GTT TTG AGG TTG AGT GAC-3′ and 5′-CAG GCT AGG GAG ATT GCA TTT CTG-3′ for *AT1R*; 5′-GGG AGC AAA TCC CAC CTT TA-3′ and 5′-GGT CAC AGT ACA ACC CAT AGTC-3′ for *mDia1*; 5′-CGA CCA CTT TGT CAA GCT CA-3′ and 5′-AGG GGA GAT TCA GTG TGG TG-3′ for *GAPDH*; and 5′-ATT CCG ATA ACG AAC GAG AC-3′ and 5′-GCT TAT GAC CCG CAC TTA CT-3′ for 18S rRNA. The mean values from triplicate (*n* = 3) measurements were used to calculate gene expression after normalization to 18S rRNA levels as the internal control.

### Measurement of HMGB1

Culture medium was concentrated from 6 to 1 ml using a centrifugal filter device (Millipore, Billerica, USA). Then, the HMGB1 levels in culture medium were determined by using an ELISA kit (Chondrex, Redmond, WA, USA) according to the manufacturer’s instructions and by western blot analysis using 20 μl of culture medium.

### In vivo study using apolipoprotein E knockout mice

Atherosclerosis-prone apolipoprotein E knockout (ApoE KO) mice, a well-established model of experimental atherosclerosis^16,17^, were used for in vivo studies. Twelve-week-old male ApoE KO mice on a C57BL/6J background were obtained from Jackson Laboratory (Bar Harbor, ME, USA). The animals were housed in a room with a 12-h light/dark cycle and were maintained in temperature-controlled clean racks for 6 weeks. All animal studies and postmortem procedures were approved by the Institutional Animal Care and Use Ethics Committee of Yonsei University (approval reference number: 2014-0350) in accordance with the NIH Guide for the Care and Use of Laboratory Animals.

ApoE KO mice were divided into the following four groups: the untreated control group [saline intraperitoneal (IP) injection], Ang II infusion group, Ang II infusion with sRAGE IP injection group, and sRAGE IP injection group. Animals were anaesthetized via IP injection of Zoletil (30 mg/kg) and Rompun (10 mg/kg), and Alzet^®^ osmotic minipumps (model 2006; DURECT Corporation, Cupertino, CA, USA) were implanted subcutaneously for the infusion of Ang II at a dosage of 1.5 μg/kg/min for 42 days. sRAGE was injected intraperitoneally daily for 42 days at a dosage of 2 μg/mouse/day.

### Evans blue administration and quantification

Animals were perfused with 5% Evans blue dye in saline through the jugular vein for 45 min. Animals were anesthetized and perfused via the left ventricle with cold PBS, and the aorta was removed. Aortas were measured and quantified using ImageJ software. Evans blue dye was eluted from the aortas by incubation with formamide at 56 °C for 2 days. The amount of dye was quantified by spectrophotometry at 610 nm.

### Transmission electron microscopy

To examine endothelial permeability, aortic tissues were fixed for 12 h in 2% glutaraldehyde–paraformaldehyde in 0.1 M phosphate buffer (pH 7.4), washed in 0.1 M phosphate buffer, and postfixed with 1% OsO_4_ dissolved in 0.1 M phosphate buffer for 2 h. Then, samples were dehydrated in ethanol and infiltrated with propylene oxide. Specimens were embedded with a Poly/Bed 812 kit (Polysciences), and 200–250-nm-thick sections were initially cut and stained with toluidine blue (T3260; Sigma-Aldrich, St. Louis, MO, USA) for light microscopy. Ultrathin sections (70 nm) were cut by a LEICA EM UC-7 (Leica Microsystems, Vienna, Austria) with a diamond knife (Diatome) and were double stained with 6% uranyl acetate (22400; Electron Microscopy Sciences, Hatfield, PA, USA) for 20 min and lead citrate (Thermo Scientific, Waltham, MA, USA) for 10 min. All sections were evaluated by transmission electron microscopy (TEM) (JEM-1011; JEOL Ltd., Tokyo, Japan) at an acceleration voltage of 80 kV.

### Statistical analysis

ImageJ 10.0, SigmaPlot, and GraphPad Prism 5.01 were used for data analysis and visualization. All data are expressed as the means ± SEMs of at least three independent experiments. Statistical comparisons were performed using Student’s *t*-test or one-way analysis of variance (ANOVA) with Tukey’s multiple comparisons test. *P* values of less than 0.05 were considered statistically significant.

## Results

### Ang II induced endothelial hyperpermeability in HUVECs

Ang II is known to induce endothelial hyperpermeability. According to our data, as the concentration of Ang II (100, 300, 500, and 1000 nM for 4 h) increased, the amount of FITC-dextran in the media in the lower chambers also increased, indicating that Ang II dose-dependently increased endothelial permeability in HUVECs (Fig. S1a). TEER is another parameter for evaluating cell permeability and is negatively correlated with cell permeability^18^. Ang II (500 nM) also significantly decreased TEER beginning at 4 h (Fig. S1b). Furthermore, the adherens junction architecture and phosphorylation state of VE-cadherin were examined to evaluate endothelial permeability^19,20^; Ang II caused the disassembly of VE-cadherin junctions (Fig. S2a) and Y731-specific phosphorylation of VE-cadherin (Fig. S2b), indicating disruption of adherens junctions. Taken together, these data indicated that Ang II induced endothelial hyperpermeability in a dose- and time-dependent manner in HUVECs.

### RAGE mediates Ang II-induced endothelial hyperpermeability

To verify the involvement of RAGE signaling in Ang II-induced endothelial hyperpermeability, siRNA specific for RAGE (Fig. S3) was used to knock down RAGE-mediated signaling. RAGE siRNA significantly suppressed the Ang II-induced upregulation of HMGB1, a major ligand for RAGE known to be upregulated by Ang II^21^, at both the mRNA (Fig. S4a) and protein (Fig. S4b) levels. During the disassembly of VE-cadherin-mediated cell–cell contacts, kinases such as p21-activated kinase (PAK), Src, and focal adhesion kinase (FAK) facilitate C-tail phosphorylation of VE-cadherin, which can lead to the dissociation of VE-cadherin from its accessory molecule β-catenin^22^.

**Fig. 1:**
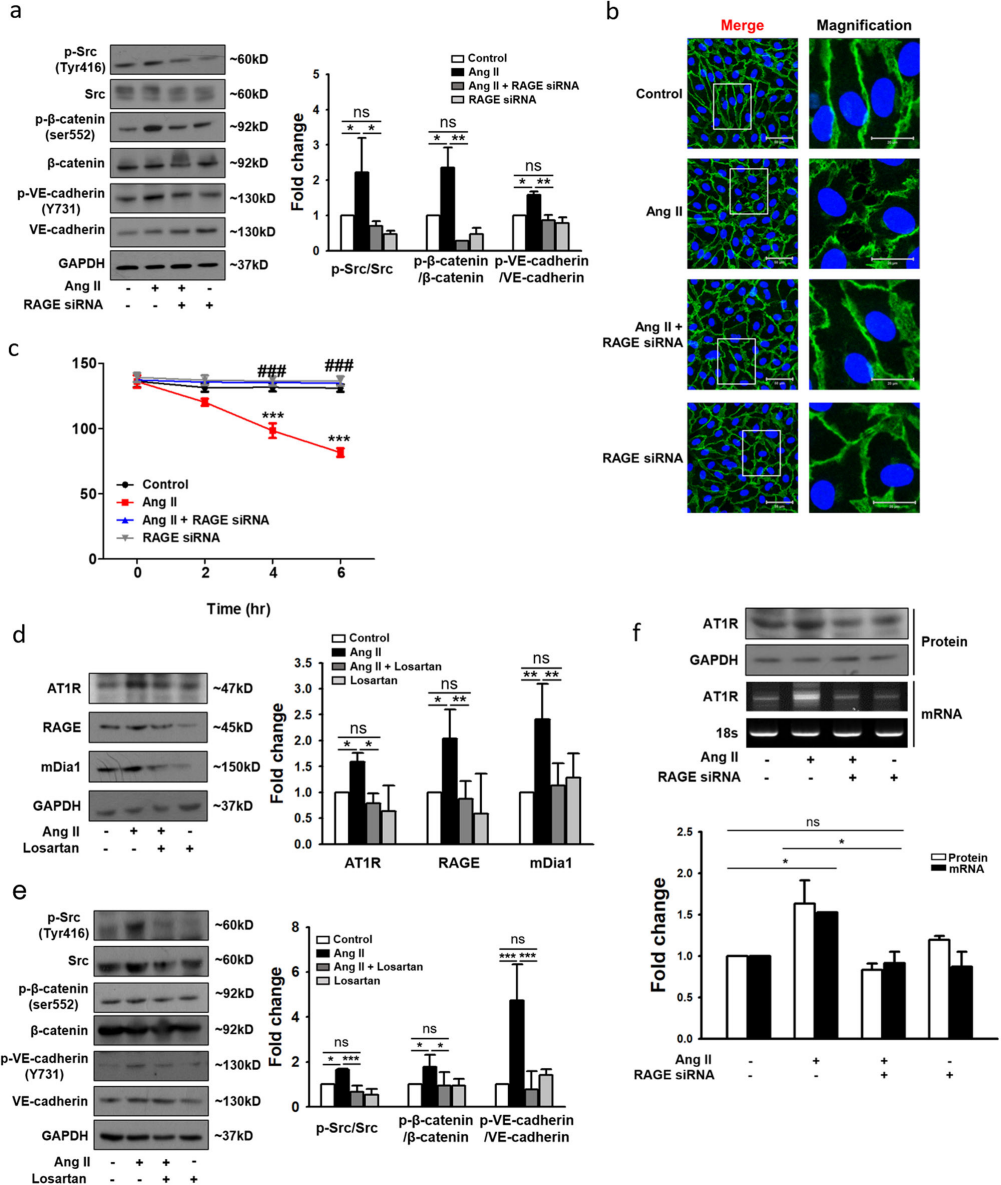
Importance of the AT1R-RAGE axis in Ang II-induced endothelial hyperpermeability in HUVECs. **a** Changes in the phosphorylation of VE-cadherin (Y731), Src (Tyr416), and β-catenin (Ser552) in HUVECs following transfection with *RAGE* siRNA and treatment with Ang II. Expression values were normalized to those of VE-cadherin, Src, and β-catenin (*n* = 4 for each lane). **b** Immunocytochemistry of VE-cadherin (green) and DAPI (nuclei, blue), as examined under a confocal microscope (white scale bars: 50 μm in merged images and 20 μm in magnified images; ×400 magnification). The main images were selected from representative regions. **c** HUVECs transfected with siRNA targeting RAGE or with scrambled sequences were incubated for 72 h and were then stimulated with Ang II for 6 h. TEER was measured every 2 h. ***p < 0.001 vs. control, ^###^*p* < 0.001 vs. Ang II (*n* = 3 for each lane). **d**, **e** Protein expression in HUVECs treated with Ang II and losartan (10 μM) alone or in combination for 4 h, as determined by western blotting. The relative values of AT1R, RAGE, and mDia1 expression values were normalized to that of GAPDH (*n* = 4 for each lane). The relative values of phospho-Src, phospho-β-catenin, and phospho-VE-cadherin expression were normalized to those of Src, β-catenin, and VE-cadherin, respectively (*n* = 3 for each lane). **f** Changes in AT1R protein levels in HUVECs following transfection with *RAGE* siRNA, as determined by western blotting. Expression was normalized to that of GAPDH (top). Changes in *AT1R* mRNA expression in HUVECs following transfection with *RAGE* siRNA, as determined by RT-PCR (bottom). Expression was normalized to that of the 18S rRNA gene (*n* = 4 for each lane). The values are presented as the means ± SEMs. *p < 0.05; **p < 0.01; ***p < 0.001; ns not significant by one-way analysis of variance (ANOVA) followed by Tukey’s multiple comparisons test

Since dephosphorylated β-catenin links cadherin to the actin cytoskeleton and thereby contributes to endothelial cell–cell junction stabilization^23^, phosphorylation of β-catenin may indicate an increased risk of VE-cadherin disassembly. According to our data, Ang II significantly increased the phosphorylation of Src, β-catenin, and VE-cadherin, but this increase was significantly attenuated by RAGE siRNA (Fig. 1a). Furthermore, RAGE siRNA attenuated the Ang II-induced disassembly of VE-cadherin (Fig. 1b) and decreased the TEER values of confluent HUVEC monolayers (Fig. 1c). These results suggested that RAGE activation modulates Ang II-induced VE-cadherin disassembly and subsequent endothelial hyperpermeability.

### Activation of AT1R augments the RAGE signaling cascade

It has been reported that AT1R stimulation by Ang II increases RAGE expression in diabetic atherosclerosis^11^, suggesting possible crosstalk between AT1R-mediated signaling and RAGE-mediated signaling. Additionally, regarding RAGE signaling in endothelial hyperpermeability, mammalian diaphanous 1 (mDia1), a member of the diaphanous-related formin family, was recently identified as a new signaling molecule in AGE-induced endothelial hyperpermeability. Mechanistically, mDia1 transduces signaling via binding to the cytoplasmic domain of RAGE (ctRAGE)^24,25^.

In the present study, Ang II significantly increased the expression of AT1R, RAGE, and mDia1, but this increase was significantly attenuated by losartan, a well-known selective, competitive AT1R antagonist^26^ (Fig. 1d). Furthermore, Ang II-induced phosphorylation of Src, β-catenin, and VE-cadherin was significantly attenuated by the AT1R antagonist (Fig. 1e). These data suggested that the activation of AT1R reinforces RAGE signaling at least partially by upregulating major mediators of RAGE signaling such as RAGE itself and mDia1. Additionally, the Ang II-induced increase in AT1R expression was significantly suppressed by RAGE siRNA at both the mRNA and protein levels (Fig. 1f), suggesting that RAGE may relay signals between AT1R activation and transcription, establishing a transactivation circuit composed of AT1R-mediated signaling and RAGE-mediated signaling.

### HMGB1 links AT1R-mediated signaling and RAGE-mediated signaling

**Fig. 2:**
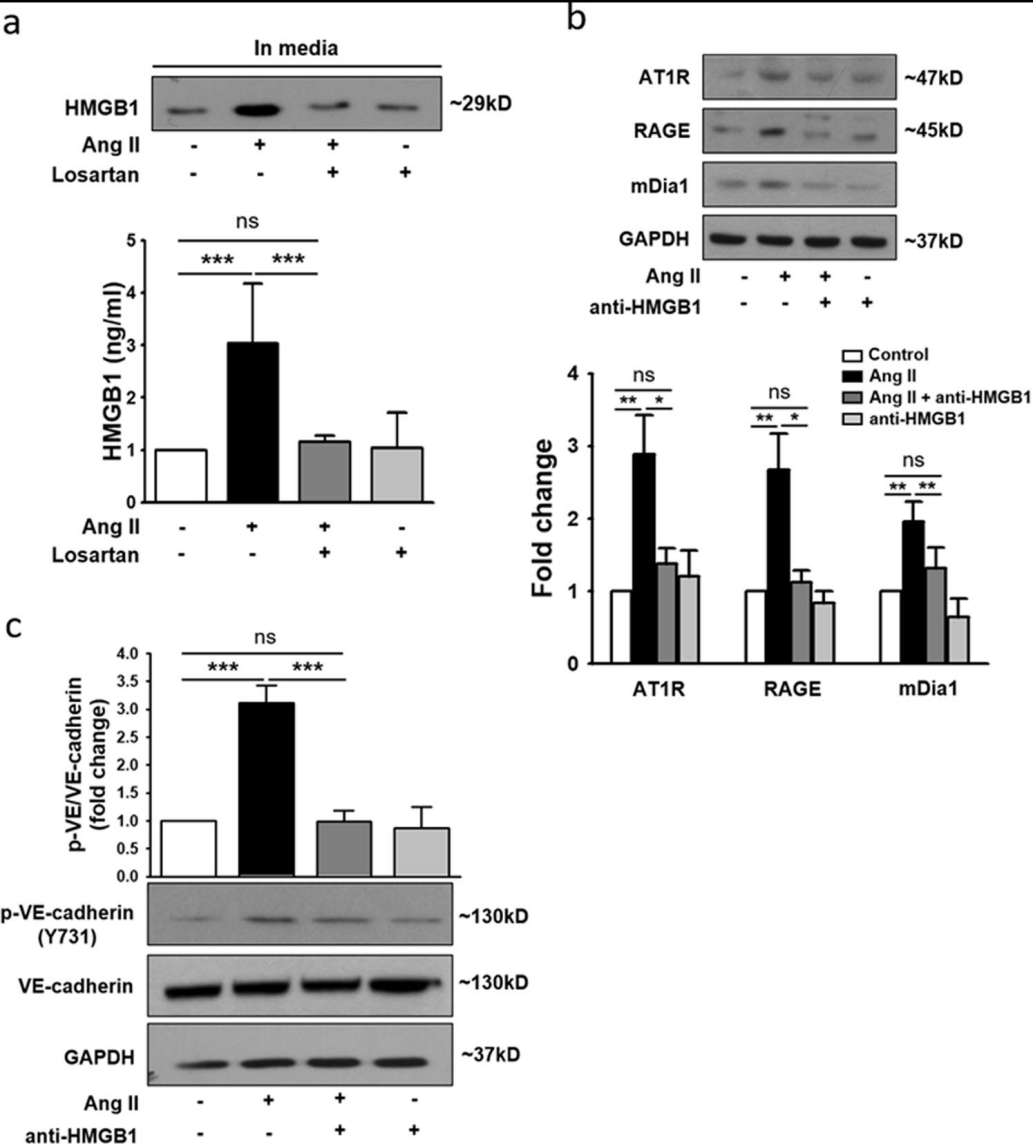
HMGB1 is an important mediator of AT1R-RAGE signaling. HUVECs were treated with Ang II in the absence or presence of losartan for 4 h. **a** HMGB1 release in supernatants was measured by ELISA and western blotting (*n* = 4 for each lane). **b**, **c** HUVECs were incubated with Ang II in the presence or absence of anti-HMGB1-neutralizing antibody (50 ng/ml) for 4 h and analyzed by western blotting. The relative values of AT1R, RAGE, and mDia1 expression were normalized to that of GAPDH (*n* = 4 for each lane). The relative values of phospho-VE-cadherin expression were normalized to that of VE-cadherin (*n* = 3 for each lane). The values are presented as the means ± SEMs. *p < 0.05; **p < 0.01; ***p < 0.001; ns not significant by one-way analysis of variance (ANOVA) followed by Tukey’s multiple comparisons test

A well-known RAGE ligand, HMGB1, can be upregulated by Ang II. In addition, our recent study demonstrated that Ang II-induced HMGB1 secretion is critical for the development of cardiac hypertrophy through RAGE activation^27^. Therefore, it was hypothesized that Ang II-induced AT1R activation increases HMGB1 secretion and that HMGB1 subsequently binds to RAGE, initiating RAGE-mediated signaling. In support of this hypothesis, the amount of HMGB1 secreted into the culture medium was significantly increased by Ang II, and this increase was inhibited by the AT1R antagonist losartan (Fig. 2a). Furthermore, HMGB1 neutralizing antibodies significantly suppressed the Ang II-induced expression of AT1R, RAGE, and mDia1 (Fig. 2b) as well as phosphorylation of VE-cadherin at Y731 (Fig. 2c). These data strongly suggested that HMGB1 is the link that connects AT1R activation with the augmentation of RAGE signaling and subsequent disassembly of VE-cadherin.

### NF-κB facilitates the key modulators of Ang II-induced endothelial hyperpermeability

**Fig. 3:**
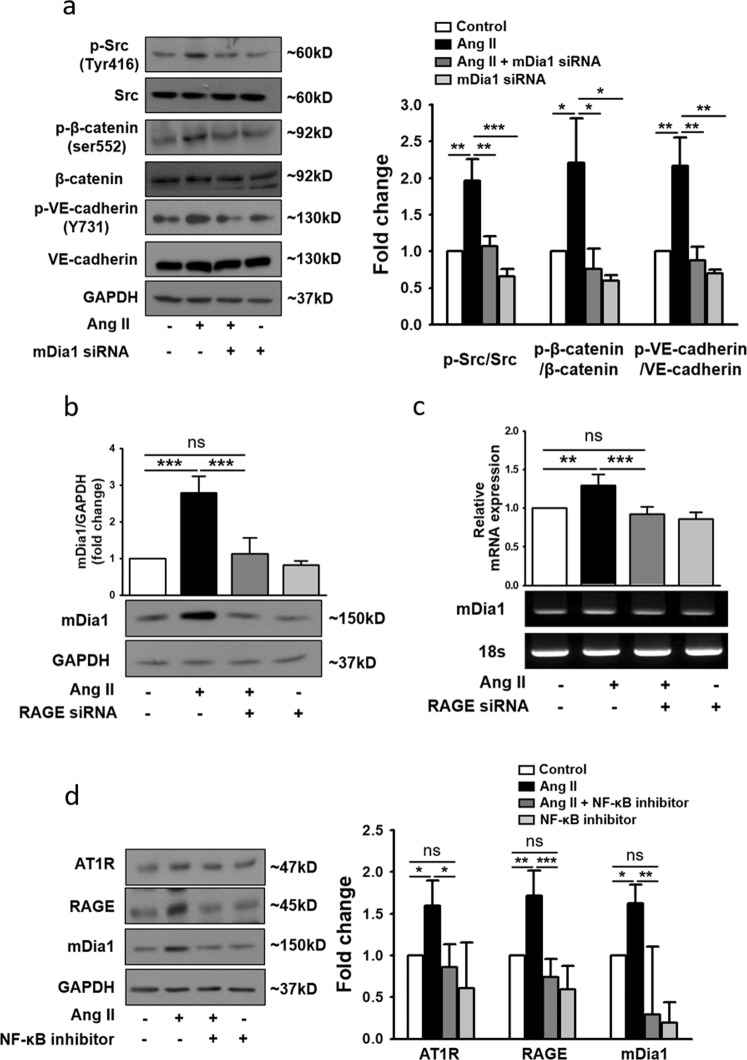
RAGE regulates Ang II-induced endothelial hyperpermeability via mDia1. **a** HUVECs were transfected with *mDia1* siRNA and cultured in the presence of Ang II for an additional 4 h. Western blot analysis was performed to observe changes in phospho-Src, phospho-β-catenin, and phospho-VE-cadherin protein expression (*n* = 4 for each lane). **b** Changes in mDia1 protein levels in HUVECs following transfection with *RAGE* siRNA, as determined by western blotting. Expression was normalized to that of GAPDH (*n* = 3 for each lane). **c** Changes in *mDia1* mRNA expression in HUVECs following transfection with *RAGE* siRNA, as determined by RT-PCR. Expression was normalized to that of the 18S rRNA gene (*n* = 4 for each lane). **d** HUVECs were treated with Ang II and the NF-κB inhibitor (5 μg/ml) alone or in combination for 4 h. Protein levels of AT1R, RAGE, and mDia1 in cell lysates were determined by western blotting. Expression was normalized to that of GAPDH (*n* = 4 for each lane). The values are presented as the means ± SEMs. *p < 0.05; **p < 0.01; ***p < 0.001; ns not significant by one-way analysis of variance (ANOVA) followed by Tukey’s multiple comparisons test

The aforementioned data indicated that Ang II augments RAGE signaling by increasing the expression of not only RAGE but also mDia1, a critical accessory molecule in the transduction of RAGE signals. To verify the importance of mDia1 in Ang II-induced endothelial hyperpermeability, mDia1-specific siRNA (Fig. S5) was utilized. mDia1 siRNA significantly attenuated Ang II-induced phosphorylation of Src, β-catenin, and VE-cadherin (Fig. 3a), demonstrating the importance of mDia1 in Ang II-induced endothelial hyperpermeability.

To further investigate the regulatory mechanism of mDia1, the effect of RAGE knockdown on mDia1 expression was examined, and the results indicated that RAGE-mediated signaling is required for Ang II-induced expression of mDia1 (Fig. 3b, c). Although the transcriptional regulation of mDia1 remains to be clarified, the transcription of both AT1R and RAGE has been reported to be mediated by the transcription factor NF-κB^28–31^. Furthermore, RAGE activation can lead to NF-κB-mediated expression of proinflammatory molecules, including RAGE itself^32^. Therefore, the effect of CAPE, a well-known NF-κB inhibitor (Fig. S6)^33^, on the expression of AT1R, RAGE, and mDia1 was examined. Similar to RAGE siRNA, CAPE significantly attenuated Ang II-induced expression of AT1R, RAGE, and mDia1 (Fig. 3d). Furthermore, CAPE significantly suppressed Ang II-induced expression of HMGB1 (Fig. S7). Taken together, these data indicated that NF-κB may be one of the major transcription factors contributing to the transcription of the key molecules involved in Ang II-induced endothelial hyperpermeability.

### sRAGE attenuates Ang II-induced endothelial hyperpermeability by disrupting the RAGE-mediated signaling cascade

Although RAGE siRNAs significantly attenuated Ang II-induced endothelial hyperpermeability in vitro, the unsolved issues with the use of siRNAs in vivo, such as their low cellular uptake, off-target effects, and instability in serum^34^, could be problematic if a RAGE signaling inhibitor were to be developed as a clinical therapeutic agent. Therefore, as an alternative to RAGE siRNA, sRAGE, which can directly act on circulating RAGE ligands such as HMGB1, was utilized to disrupt RAGE signaling. According to our data, sRAGE significantly attenuated the Ang II-induced decrease in TEER in a dose- and time-dependent manner (Fig. S8), suggesting that it was effective in suppressing the induction of endothelial hyperpermeability by Ang II.

**Fig. 4:**
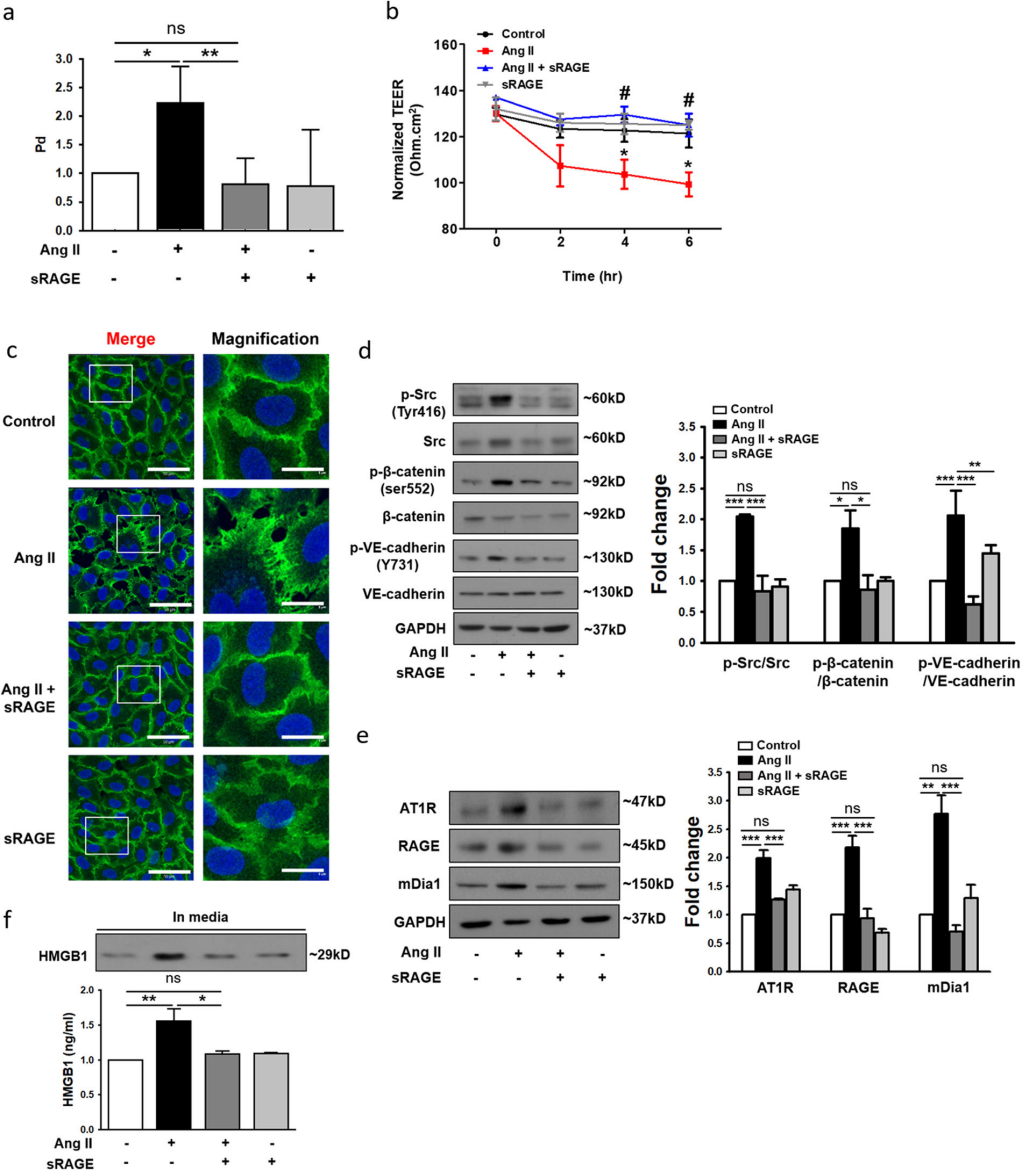
Soluble RAGE (sRAGE) attenuates Ang II-induced endothelial hyperpermeability. **a** HUVECs were treated with sRAGE (2 μg/ml) for 1 h and were then subjected to Ang II treatment for 4 h. Endothelial permeability was assessed in media from the lower chambers after the addition of FITC-dextran 40 to the upper chambers for 1 h (*n* = 3 for each lane). **b** TEER was measured every 2 h. *p < 0.05 vs. control, ^#^*p* < 0.05 vs. Ang II (*n* = 3 for each lane). **c** Immunocytochemistry of VE-cadherin (green) and DAPI (nuclei, blue) was performed, and samples were directly examined under a confocal microscope (white scale bars: 50 μm in merged images and 5 μm in magnified images; ×400 magnification). The main images were selected from representative regions. **d** HUVEC extracts were analyzed by western blotting to assess phospho-Src, phospho-β-catenin, and phospho-VE-cadherin expression. Expression values were normalized to those of Src, β-catenin, and VE-cadherin (*n* = 4 for each lane). **e** The protein levels of AT1R, RAGE, and mDia1 were determined by western blotting. Expression was normalized to that of GAPDH (*n* = 3 for each lane). **f** HMGB1 release in supernatants was measured by ELISA and western blotting (*n* = 3 for each lane). The values are presented as the means ± SEMs. *p < 0.05; **p < 0.01; ***p < 0.001; ns not significant by one-way analysis of variance (ANOVA) followed by Tukey’s multiple comparisons test

When sRAGE was used alone at a concentration of 2 µg/ml, it did not significantly affect the permeability of the endothelial monolayer but significantly attenuated the Ang II-induced increase in the permeability of the endothelial monolayer, as evidenced by the results of the permeability assay using FITC-labeled dextran 40 (Fig. 4a) and the TEER assay (Fig. 4b). The immunocytochemical analysis results showed that sRAGE also prevented Ang II-induced dissociation of VE-cadherin (Fig. 4c) and phosphorylation of Src, β-catenin, and VE-cadherin (Fig. 4d). Furthermore, Ang II-induced upregulation of AT1R, RAGE, and mDia1 was significantly attenuated by sRAGE at both the mRNA level (Fig. S9) and the protein level (Fig. 4e). The amount of HMGB1 secreted following Ang II stimulation was also decreased by sRAGE treatment (Fig. 4f), possibly due to the suppression of Ang II-induced HMGB1 mRNA synthesis (Fig. S10). These results indicated that the effect of sRAGE was comparable to that of RAGE siRNA in terms of both disrupting the RAGE-mediated signaling cascade and preventing Ang II-induced endothelial hyperpermeability in vitro.

### In vivo delivery of sRAGE attenuates Ang II-induced endothelial hyperpermeability of the aorta in ApoE KO mice

**Fig. 5:**
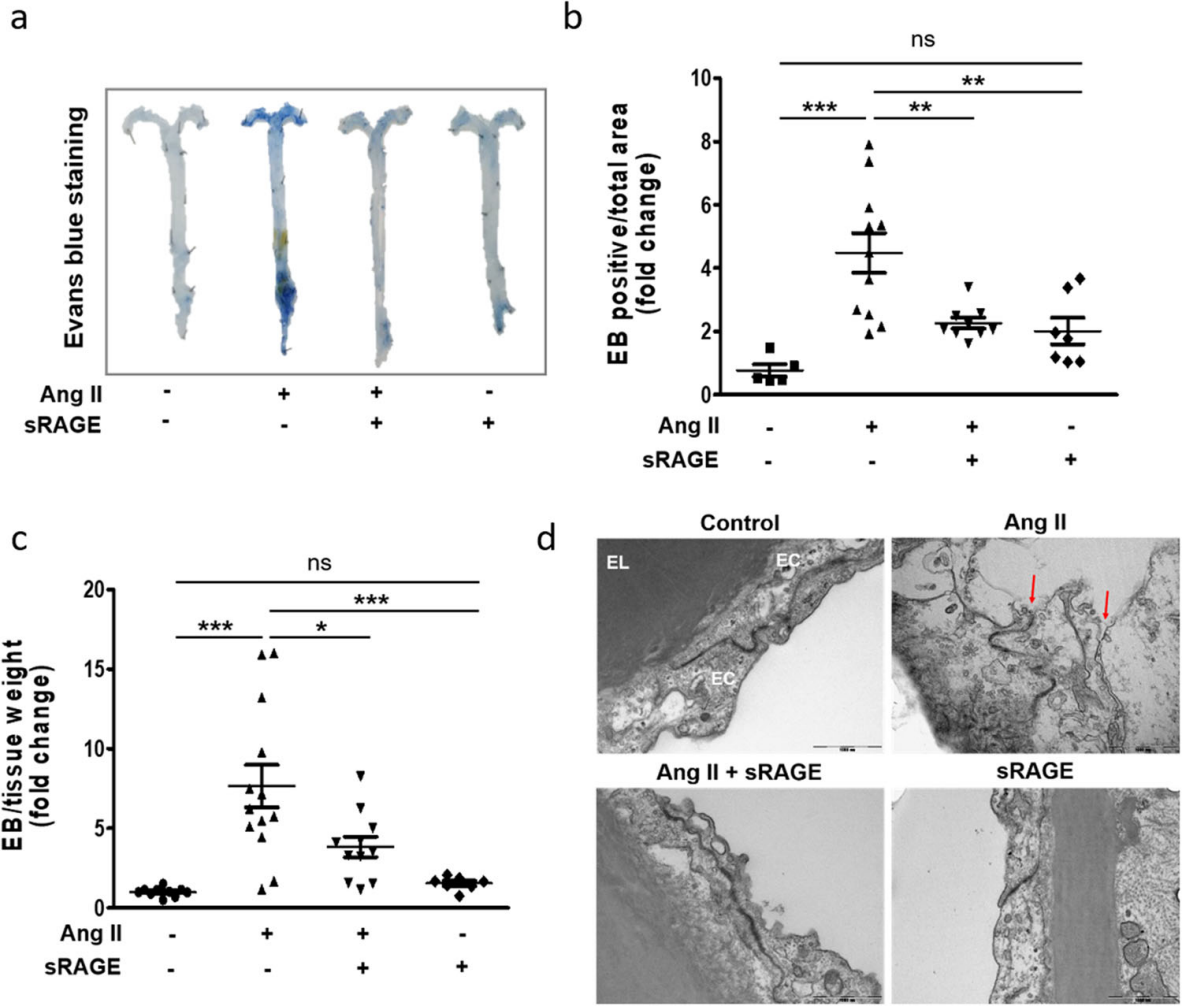
Effects of sRAGE on Ang II-induced endothelial hyperpermeability in vivo. Twelve-week-old ApoE KO mice were used to investigate endothelial hyperpermeability. Ang II was injected into mice with or without sRAGE for 6 weeks. Then, Evans blue (EB) dye in saline was administered via the jugular vein. **a** Representative photographs of aortas stained with Evans blue in ApoE KO mice. **b** Quantification of positive areas of Evans blue staining in the aortas, as estimated using ImageJ. **c** Evans blue dye was eluted from the aortas by incubation with formamide. The amount of dye was quantified by spectrophotometry at 610 nm. **d** Representative TEM images of the EC junction area (original magnification, ×30,000). EL elastic lamina, EC endothelial cell; scale bar, 1000 nm. Control group, *n* = 10; Ang II group, *n* = 13; Ang II + sRAGE group, *n* = 11; sRAGE group, *n* = 7. The results are representative of at least four separate experiments. The values are presented as the means ± SEMs. *p < 0.05; **p < 0.01; ***p < 0.001; ns not significant by one-way analysis of variance (ANOVA) followed by Tukey’s multiple comparison test

The Evans blue leakage assay is a well-accepted method for evaluating vascular permeability^35^. As shown in Fig. 5a, vascular permeability was prominently increased by Ang II infusion compared to that in the control group, whereas in vivo delivery of sRAGE dramatically attenuated Ang II-induced vascular hyperpermeability. This finding was quantitatively confirmed by calculating both the Evans blue-positive area with respect to the total area (Fig. 5b) and the total amount of Evans blue per gram of tissue (Fig. 5c). Closer examination of the endothelium using TEM demonstrated that Ang II infusion caused widening of the intercellular space, which was prevented by in vivo sRAGE delivery (Fig. 5d). Taken together, these results indicated that in vivo delivery of sRAGE effectively suppressed Ang II-induced vascular hyperpermeability in vivo.

## Discussion

Previous studies have demonstrated that both Ang II signaling and RAGE-mediated signaling play important roles in the development of endothelial hyperpermeability^15,36^. Although the importance of crosstalk between signaling through AT1R and RAGE in the pathogenesis of diabetic atherosclerosis has been reported^11^, to our knowledge, such crosstalk between these two signaling pathways in the mediation of endothelial hyperpermeability has not been experimentally confirmed. In the present study, we provided empirical evidence that these two pathways are linked by demonstrating that Ang II increased the production of HMGB1, one of the major ligands for RAGE, in an NF-κB-dependent manner (Fig. 2a, Fig. S7) and that the neutralization of secreted HMGB1 using either HMGB1-specific antibodies (Fig. 2b, c) or sRAGE, a decoy receptor for HMGB1 (Figs. 4 and 5), significantly attenuated Ang II-induced endothelial hyperpermeability. Thus, the present study is the first to report the existence of HMGB1-mediated crosstalk between Ang II signaling and RAGE-mediated signaling in endothelial hyperpermeability.

One of the important physiological functions of the vascular endothelium is to regulate exchange between the blood and interstitial fluid, and meticulous regulation of endothelial permeability is critical for the maintenance of circulatory homeostasis^2^, considering that endothelial dysfunction or endothelial hyperpermeability can exacerbate a variety of diseases, including but not limited to cardiovascular diseases and tumor metastasis^37,38^. To date, several mechanisms of endothelial permeability regulation have been proposed, and Ang II is one of the well-established agents that causes endothelial hyperpermeability^39–41^. Consistent with these previous results, Ang II significantly induced endothelial hyperpermeability, demonstrating the disruption of endothelial cell adherens junctions, a common characteristic of endothelial hyperpermeability (Fig. S2).

VE-cadherin is a component of endothelial adherens junctions that plays a major role in the maintenance of endothelial cell–cell contact by interacting with the cytoskeleton via anchoring molecules such as β-catenin^19,42^, and its physiological regulation is compromised in Ang II-induced endothelial dysfunction^43,44^. The cytoplasmic tail of VE-cadherin contains at least five tyrosine residues (Y648, Y658, Y685, Y731, and Y733), and phosphorylation of Y731 mediated by the cytoplasmic protein tyrosine kinase Src is especially important in modulating VE-cadherin activity, since C-tail phosphorylation of VE-cadherin can lead to the dissociation of VE-cadherin from its accessory molecule β-catenin^22,45^. Furthermore, dephosphorylated β-catenin stabilizes endothelial cell–cell junctions by linking cadherin to the actin cytoskeleton^23^; therefore, phosphorylation of β-catenin may indicate an increased risk of VE-cadherin disassembly and subsequent endothelial hyperpermeability^46,47^.

In the present study, by demonstrating that knockdown of RAGE via siRNA effectively suppressed Ang II-induced phosphorylation of Src, β-catenin, and VE-cadherin (Fig. 1a) as well as the disruption of adherens junctions (Fig. 1b) and the decrease in TEER (Fig. 1c), we proved that the RAGE-mediated signaling cascade contributes significantly to Ang II-induced endothelial hyperpermeability. It has been reported that RAGE-mediated signal transduction requires interaction of the RAGE cytoplasmic domain with the FH1 domain of the formin mDia1 (refs. ^25,48^). Furthermore, according to our data, RAGE not only requires mDia1 to transmit the signal downstream (Fig. 3a) but also positively regulates the expression of mDia1 in an NF-κB-dependent manner (Fig. 3d), suggesting a positive feedback loop between RAGE and mDia1 to augment the RAGE-mediated signaling cascade. In fact, since the NF-κB inhibitor also attenuated Ang II-induced expression of AT1R and RAGE (Fig. 3d), it was speculated that NF-κB was a key molecule facilitating an extended positive feedback network involving AT1R, RAGE, and mDia1 in Ang II-induced endothelial hyperpermeability. Previous studies demonstrating RAGE-mediated activation of NF-κB^27,49^ and NF-κB-dependent upregulation of AT1R^28,31^ and RAGE^29,30^ also support this speculation. However, without direct empirical evidence, this speculation has to remain a hypothesis yet to be tested, and this insufficiency is one of the limitations of the present study.

Assuming that NF-κB is the key intracellular mediator of Ang II-induced endothelial hyperpermeability, HMGB1 seems to be the critical link that connects AT1R-mediated signaling and RAGE-mediated signaling in the present study. Although HMGB1 is a nonhistone nuclear protein primarily localized in the nucleus^50^, it can be actively released into the extracellular space upon exposure to inflammatory stimuli such as TNF-α^51^. Additionally—and more closely related to the present study—Ang II-induced upregulation of HMGB1 has also been reported^27,52^. These previous observations agree well with our data that clearly demonstrated AT1R-mediated upregulation of HMGB1 (Fig. 2a). Furthermore, yet another previous study reported HMGB1-induced endothelial hyperpermeability^53,54^, and our data demonstrating the effect of the HMGB1-neutralizing antibody on suppressing the upregulation of key molecules (Fig. 2a) and VE-cadherin phosphorylation (Fig. 2c) strongly support the idea that HMGB1 is the critical link in Ang II-induced endothelial hyperpermeability. However, the observation that both RAGE siRNA (Fig. S4) and the NF-κB inhibitor (Fig. S7) significantly suppressed Ang II-induced upregulation of HMGB1 strongly implies that Ang II-induced upregulation of HMGB1 was achieved not only by AT1R activation but also by RAGE activation. Multiple steps or multiple mechanisms encompassing both AT1R activation and RAGE activation might have been acting to generate the Ang II-induced upregulation of HMGB1 observed in the present study. Based on the acquired data, the following stepwise signaling events are speculated to occur.

**Fig. 6:**
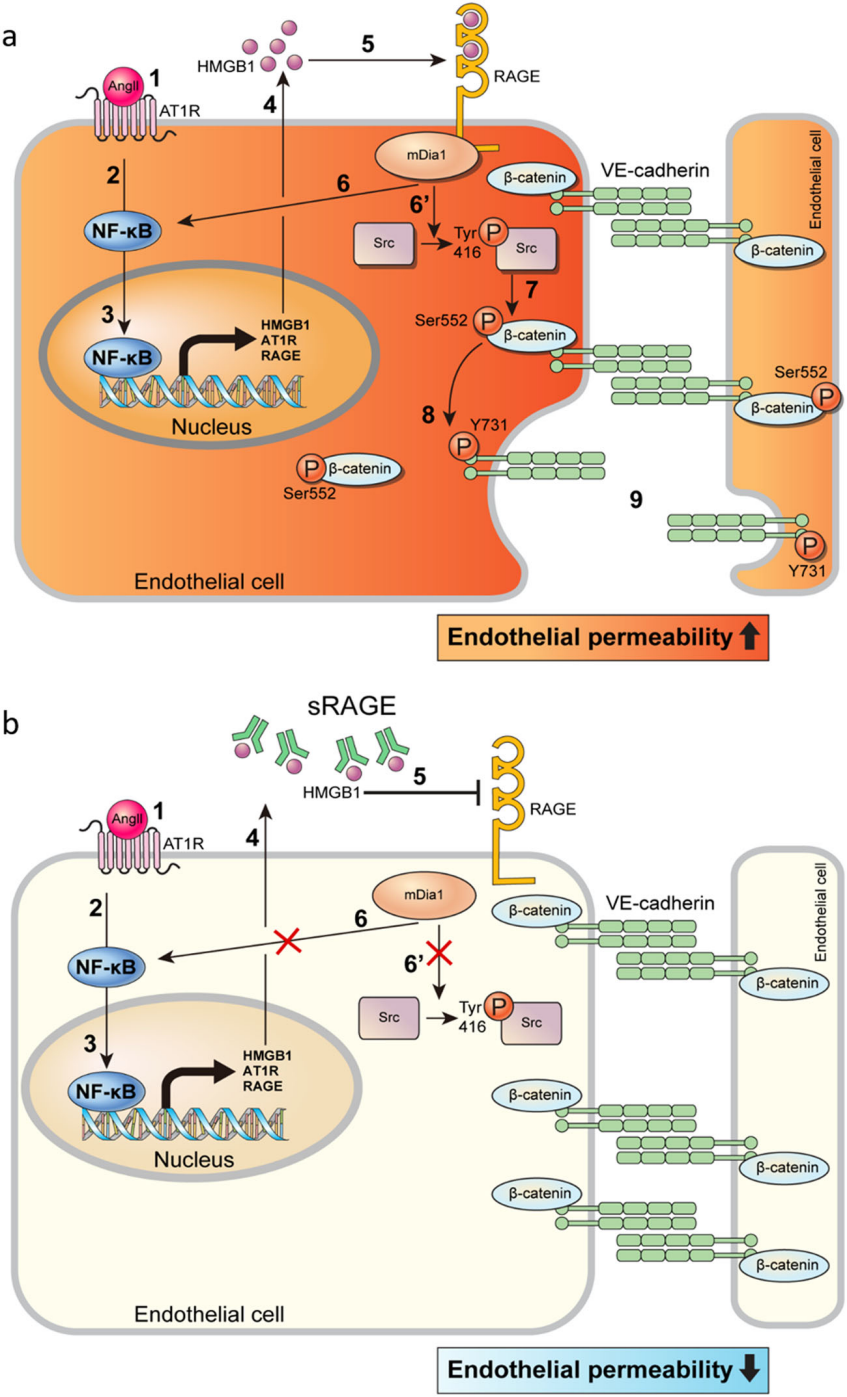
Schematic diagram of signal transduction pathways involved in Ang II-induced endothelial hyperpermeability via AT1R/RAGE/mDia1/Src/β-catenin/VE-cadherin. **a** As Ang II binds to and stimulates AT1R, the expression and secretion of HMGB1 can be increased by NF-κB activation. NF-κB-mediated expression of proinflammatory molecules, including RAGE itself, can occur. HMGB1 binds to RAGE, which can induce RAGE-mediated activation of Src/β-catenin/VE-cadherin via mDia1. **b** Blockade of RAGE activation by sRAGE attenuates the Ang II-induced increase in endothelial hyperpermeability by inhibiting RAGE-mediated signaling pathways

First, Ang II activates AT1R, which subsequently activates NF-κB. Activated NF-κB then increases the expression of HMGB1 along with that of AT1R, RAGE, and mDia1. The increased HMGB1 is then released into the extracellular space to activate RAGE. Finally, activated RAGE subsequently reactivates NF-κB to further increase HMGB1 expression. In this scenario, Ang II can directly increase the expression of HMGB1 by activating NF-κB at the early stage (step 1 in Fig. 6) and indirectly increase the expression of HMGB1 later by activating RAGE (step 4 in Fig. 6). Therefore, blocking RAGE signaling via siRNA or NF-κB inhibition could at least partially suppress Ang II-induced upregulation of HMGB1. Additionally, Ang II-induced hyperacetylation of HMGB1 might cooperate with the above mechanism to enhance the release of HMGB1 following Ang II stimulation^55^. However, these proposed mechanisms were not examined in detail in the present study, another major limitation of this study. A systematic elucidation of the underlying mechanisms, including the NF-κB-dependent upregulation of key molecules mentioned above, would make a very interesting subject for further studies.

As our data demonstrate, knockdown of the RAGE signaling cascade using RAGE-specific siRNA effectively attenuated Ang II-induced endothelial hyperpermeability in vitro (Fig. 1a–c). However, there is concern about using siRNAs in in vivo studies, although using siRNA in vivo would have been ideal for the present study. In contrast to in vitro experiments in which most variables are controllable, in vivo conditions are difficult to control due to the excessive number of uncontrollable variables. Furthermore, especially for siRNA-mediated knockdown in vivo, unsolved issues such as the low cellular uptake, off-target effects, and instability in serum still exist^34^. By using RAGE knockout animals^14^ instead of siRNA-mediated knockdown, these limitations could have been avoided. However, unfortunately, this animal model exceeded the budget; thus, sRAGE was utilized as the second-best option in the present study. Since sRAGE is expected to act on circulating RAGE ligands such as HMGB1, delivering sRAGE via IP injection was thought to be a much more reliable approach than RAGE knockdown using siRNA, which has to first enter endothelial cells to exert the expected effect. Thus, if a RAGE signaling inhibitor were to be developed as a clinical therapeutic agent, delivering peptides would be more effective than delivering siRNA. Additionally, since the main aim of the present study was to suppress RAGE-mediated signaling rather than the expression of RAGE itself, we believe that using sRAGE rather than siRNA sufficiently serves the purpose and is well justified.

sRAGE is a matrix metalloproteinase-cleaved form of RAGE released from the cell surface that functions as an extracellular decoy for RAGE ligands; sRAGE has been implicated in chronic diseases and suggested to be a therapeutically useful biomarker for diseases involving the activation of RAGE signaling cascades^56,57^. The therapeutic potential of sRAGE has been demonstrated in various in vivo disease models, including models of vascular inflammation, diabetes, retinal vascular/neuronal dysfunction, and cardiomyocyte hypertrophy^27,58,59^. However, to our knowledge, its therapeutic potential in Ang II-induced endothelial hyperpermeability has not been empirically examined. Therefore, the present study is the first to investigate the therapeutic potential of sRAGE in the prevention of endothelial hyperpermeability. According to our data, sRAGE effectively attenuated Ang II-induced endothelial permeability in vitro (Fig. 4). Additionally, in the in vivo study using Ang II-infused atherosclerosis-prone apolipoprotein E knockout mice, sRAGE significantly decreased the Evans blue-positive areas compared to those in the Ang II-infused or the saline-infused group, effectively maintaining the integrity of cell–cell contacts (Fig. 5).

The present study provided strong evidence for HMGB1-mediated crosstalk between AT1R-mediated signaling cascades and RAGE-mediated signaling cascades in Ang II-induced endothelial hyperpermeability (Fig. 6). In the clinical context, the results of this study indicate that disrupting the crosstalk between AT1R signaling and RAGE signaling can be an effective means to control Ang II-induced endothelial dysfunction and that sRAGE-mediated blockade of RAGE signaling has significant therapeutic potential. To fully address the unanswered issues, further studies are warranted.

## Supplementary information

Supplementary data
