# Supplementary material for: Soluble RAGE attenuates AngII-induced endothelial hyperpermeability by disrupting HMGB1-mediated crosstalk between AT1R and RAGE
Source: Exp Mol Med. 2019 Sep 27;51(9):113. doi: 10.1038/s12276-019-0312-5 (PMC6802637; doi:10.1038/s12276-019-0312-5)

**Supplemental Materials**

**Soluble RAGE attenuates AngII-induced endothelial hyperpermeability by disrupting HMGB1-mediated crosstalk between AT1R and RAGE**

Jisu Jeong^1,2^, Jiye Lee^2^, Juyeon Lim^1,2^, Soyoung Cho^1,2^, Soyoung An^2^, Myungeun Lee^2^, Nara Yoon^3^, Miran Seo^2^, Soyeon Lim^4*^, Sungha Park^2,5*^

1. Graduate Program in Science for Aging, Yonsei University, Seoul 120-752, Republic of Korea

2. Integrative Research Center for Cerebrovascular and Cardiovascular diseases, Yonsei University College of Medicine, Seoul 120-752, Korea

3. Department of Pathology, The Catholic University of Korea, Incheon St. Mary’s Hospital, Incheon, Korea

4. Institute for Bio-Medical Convergence, College of Medicine, Catholic Kwandong University, Gangneung, Gangwon-do 25601, Republic of Korea

5. Cardiovascular Research Institute, Division of Cardiology, Yonsei University College of Medicine, Seoul 120-752, Korea

**Subtitle**: Role of sRAGE in endothelial barrier dysfunction

**Correspondence to** Soyeon Lim, PhD, Catholic Kwandong University International St. Mary's Hospital, Incheon 22711, Republic of Korea (E mail: [slim724@cku.ac.kr/](mailto:slim724@cku.ac.kr/) 82-32-290-2777) or Sungha Park, MD/PhD, Yonsei University College of Medicine; Division of Cardiology, Yonsei University College of Medicine, Seoul 120-752, Korea (E mail: [shpark0530@yuhs.ac/](mailto:shpark0530@yuhs.ac/) 82-2-2228-8455)

**Supplemental Figures**

**Figure S1. Effects of Ang II on endothelial permeability in HUVECs**

(a) HUVECs were treated with Ang II at a concentration of 100, 300, 500, or 1000 nM for 4 h. Permeability coefficient of the transflux of tracer FITC-dextran (Pd) was measured (n=4 for each lane). (b) HUVECs were treated with 500 nM of Ang II and TEER was measured every 2 h (n=3 for each lane). Values are presented as means ± SEM. *p<0.05, **p<0.01 vs control for one-way analysis of variance (ANOVA) followed by Tukey’s multiple comparison test.


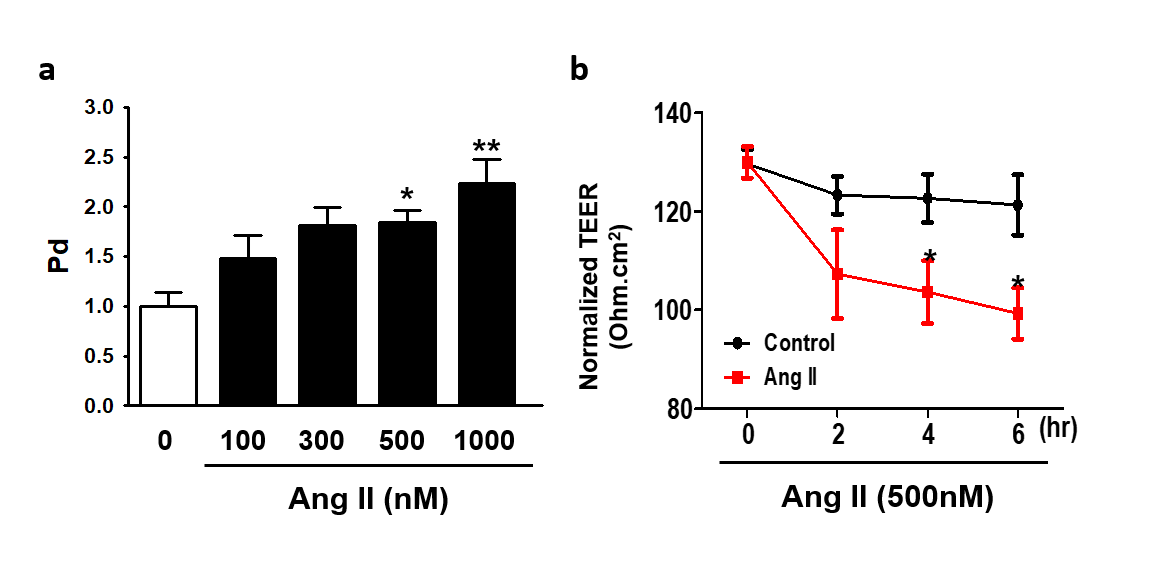


**Figure S2. Ang II induced disruption of adherens junctions and phosphorylation of VE-cadherin**

(a) Immunocytochemistry of VE-cadherin (green) and DAPI (nuclei, blue) was performed, and samples were directly examined under a confocal microscope (white scale bar: 50 μm, ×400 magnification). Main images are selected from representative regions from the experiments. (b) HUVECs were incubated with Ang II for 4 h, and then phosphorylation of VE-cadherin was measured by western blotting. The values of the relative densities of p-VE-cadherin Y685 and Y731 bands were normalized to that of VE-cadherin (n=3 for each lane). Results are representative of at least four separate experiments. Values are means ± SEM, *p<0.05 vs control, *ns* not significant, analysis was performed with an unpaired Student’s t test.


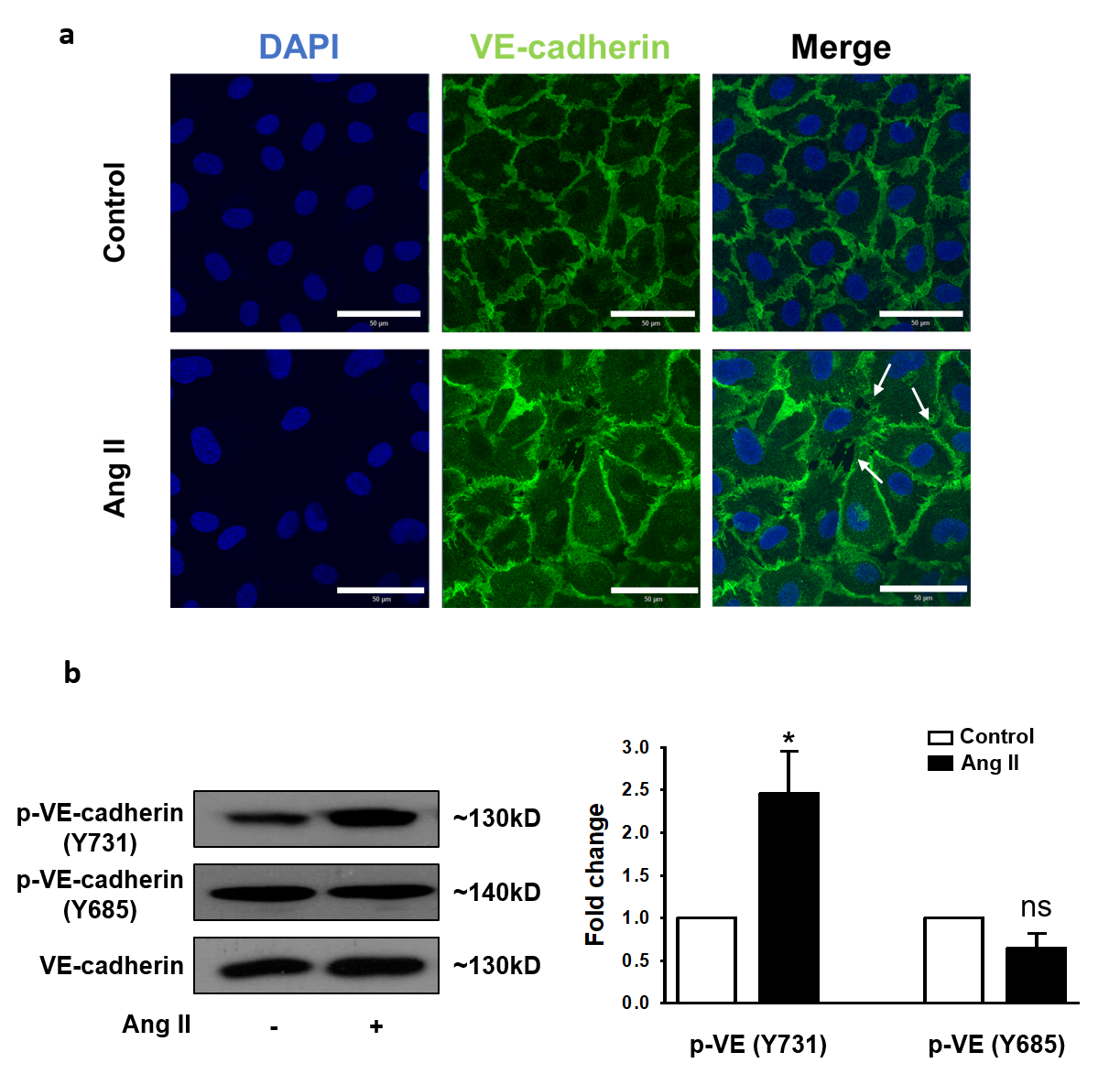


**Figure S3. Knock down of RAGE in HUVECs**

RAGE expression after transfection of HUVECs with *RAGE* siRNA for 72 h and then stimulation with 500 nM Ang II for an additional 4 h. Relative values were normalized to that of *GAPDH* (n=5 for each lane). Values are presented as means ± SEM. *p<0.05, **p<0.01, ***p<0.001 for one-way analysis of variance (ANOVA) followed by Tukey’s multiple comparison test.


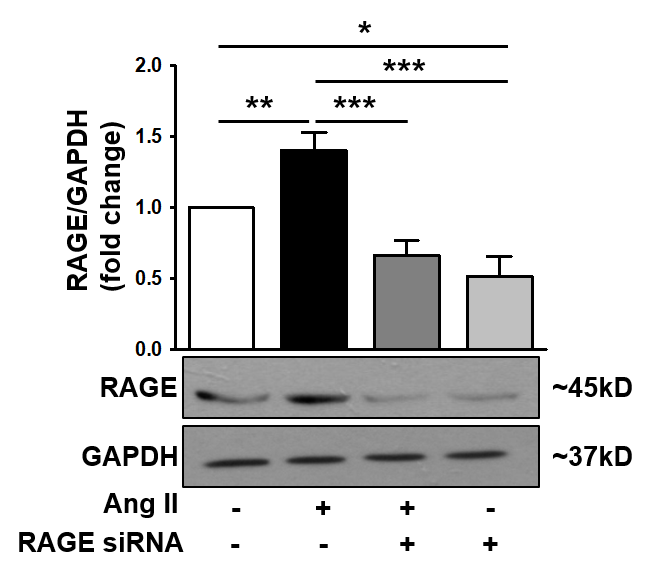


**Figure S4. Regulation of HMGB1 expression level in HUVECs.**

(a) HUVECs were transfected with *RAGE* siRNA for 72 h and then stimulated with Ang II for an additional 4 h. *HMGB1* mRNA expression was detected by RT-PCR and normalized to that of the 18S rRNA gene (n=3 for each lane). (b) Western blotting analysis of HMGB1 normalized to GAPDH expression (n=3 for each lane). Values are presented as means ± SEM. *p<0.05, **p<0.01, ***p<0.001, *ns* not significant for one-way analysis of variance (ANOVA) followed by Tukey’s multiple comparison test.


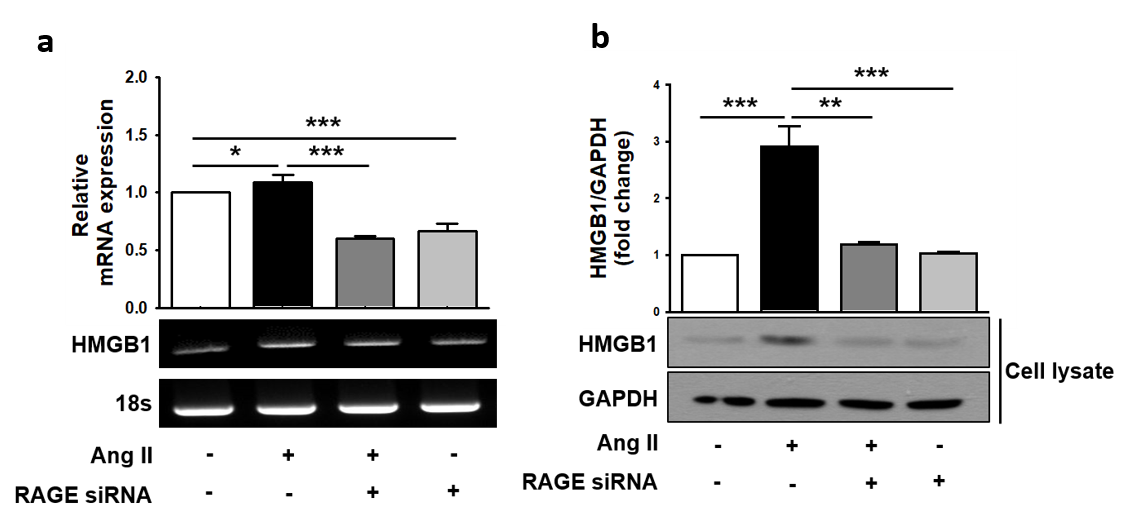


**Figure S5. Knock down of mDia1 in HUVECs**

HUVECs were transfected with *mDia1* siRNA for 48 h and cultured in the presence of Ang II for an additional 4 h. Relative values were normalized to that of *GAPDH* (n=3 for each lane). Values are presented as means ± SEM. *p<0.05, ***p<0.001 for one-way analysis of variance (ANOVA) followed by Tukey’s multiple comparison test.


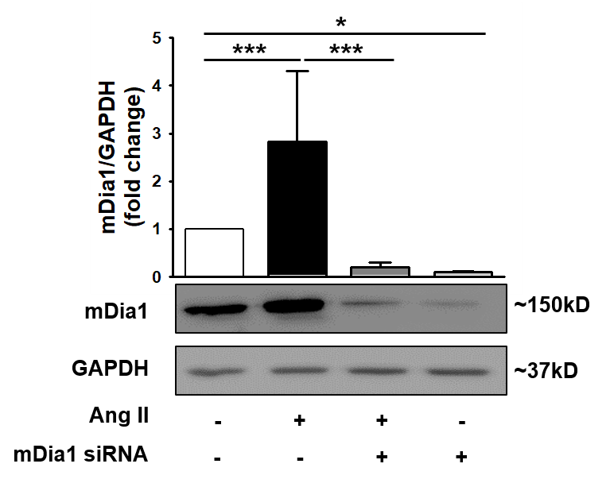


**Figure S6. Relative protein levels of p-NF-κB and NF-κB in HUVECs.**

HUVECs were pretreated with NF-κB inhibitor for 1 h and then stimulated with Ang II for an additional 4 h, and analyzed by western blotting. Values of relative densities of p-NF-κB band were normalized to density of NF-κB (n=3 for each lane). Values are means ± SEM, *p<0.05 for one-way analysis of variance (ANOVA) followed by Tukey’s multiple comparison test.


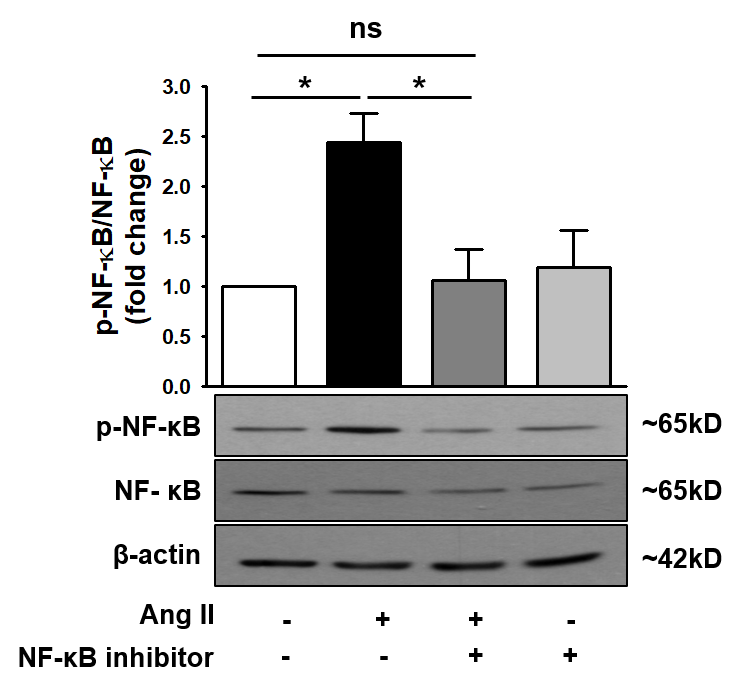


**Figure S7. Regulation of HMGB1 expression level by NF-κB in HUVECs.**

HUVECs were pretreated with NF-κB inhibitor for 1 h and then stimulated with Ang II for an additional 4 h. Protein levels of HMGB1 in cell lysates were detected by western blotting. Expression was normalized to that of GAPDH (n=4 for each lane). Values are presented as means ± SEM. ***p<0.001, *ns* not significant for one-way analysis of variance (ANOVA) followed by Tukey’s multiple comparison test.


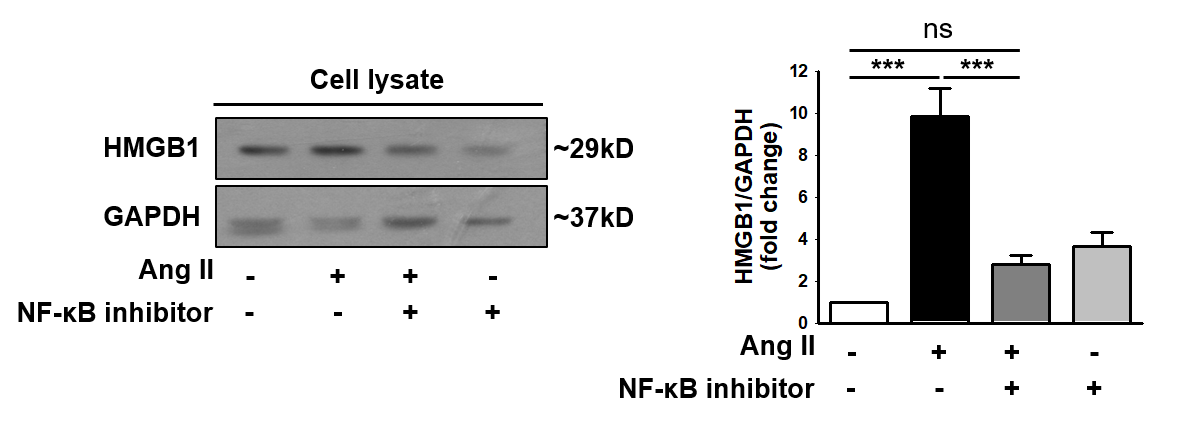


**Figure S8. Dose-dependent effects of sRAGE on Ang II-induced endothelial hyperpermeability in HUVECs**

HUVECs were treated with Ang II with or without sRAGE at 0.1, 0.5, 1, 2 μg/ml for 6 h. TEER was measured every 2 h (n=3 for each lane). Values are presented as means ± SEM. ***p<0.001 vs control, ^#^p<0.05, ^##^p<0.01, ^###^p<0.001 vs Ang II, analysis was performed with an unpaired Student’s t test.


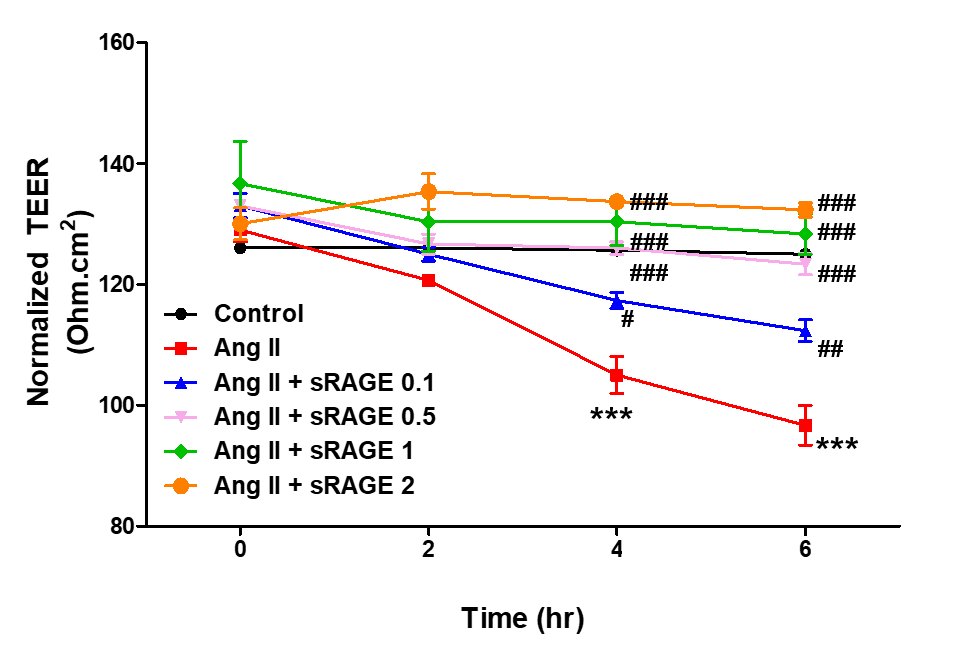


**Figure S9. sRAGE attenuates Ang II-induced AT1R, RAGE and mDia1 expression**

HUVECs were pretreated with sRAGE for 1 h and then stimulated with Ang II for an additional 4 h. *AT1R*, *RAGE*, and *mDia1* mRNA expressions were detected by RT-PCR and normalized to that of the 18S rRNA gene (n=5 for each lane). Values are means ± SEM, *p<0.05 for one-way analysis of variance (ANOVA) followed by Tukey’s multiple comparison test.


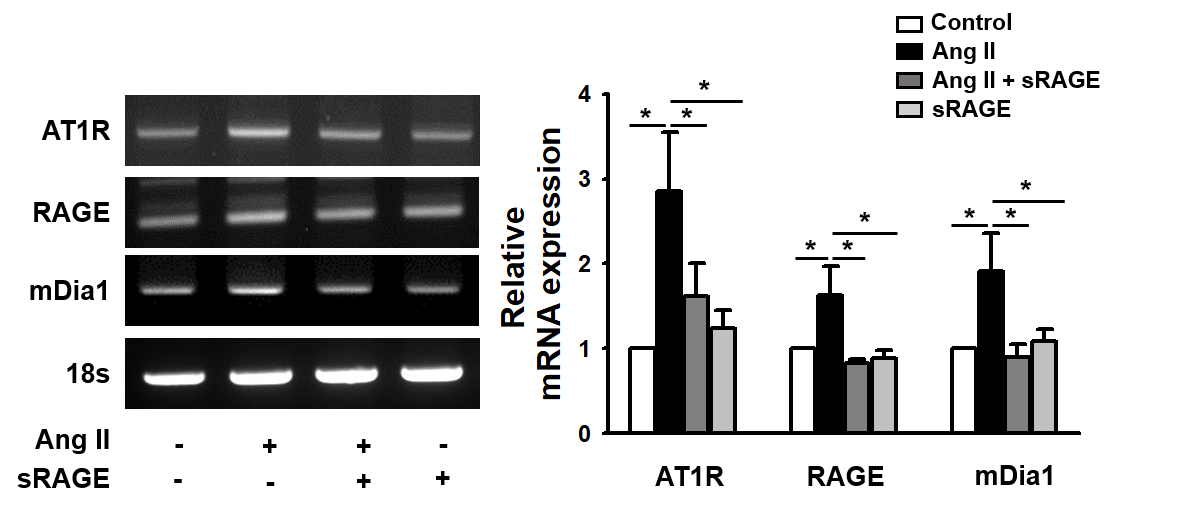


**Figure S10. sRAGE attenuates Ang II-induced HMGB1 expression**

HUVECs were pretreated with sRAGE for 1 h and then stimulated with Ang II for an additional 4 h. HMGB1 expressions was detected by RT-PCR and normalized to that of the 18S rRNA gene (n=3 for each lane). Values are means ± SEM, *p<0.05, ***p<0.001 for one-way analysis of variance (ANOVA) followed by Tukey’s multiple comparison test.


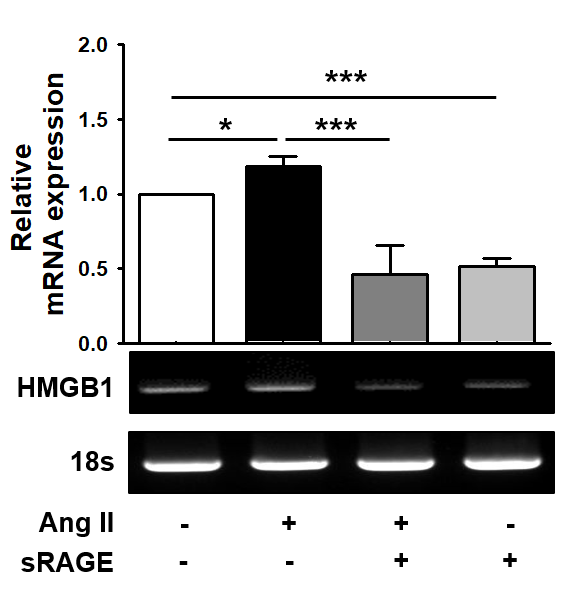

Supplement: Supplementary file 1 — Supplementary data [file 12276_2019_312_MOESM1_ESM.docx]
